# Supplementary material for: RespiCellTM: An Innovative Dissolution Apparatus for Inhaled Products
Source: Pharmaceutics. 2021 Sep 23;13(10):1541. doi: 10.3390/pharmaceutics13101541 (PMC8540329; doi:10.3390/pharmaceutics13101541)
Supplement: Supplementary file 1 [file pharmaceutics-13-01541-s001.zip › pharmaceutics-1358856-sm-XML-revised.pdf]

pharmaceutics MDPI

Supplementary Material: RespiCell™: An Innovative Dissolution Apparatus for Inhaled Products

Fabio Sonvico, Veronica Chierici, Giada Varacca, Eride Quarta, Davide D'Angelo, Ben Forbes and Francesca Buttini

Table S1. Active Pharmacological Ingredients Aqueous Solubilities.

API	Solubility in Water
NCE-A	0.157 µg/mL
NCE-B	< 0.1 µg/mL
Tobramycin	1000 mg/mL
Tiotropium HBr	25 mg/mL
Indacaterol Maleate	0.8 µg/mL

Simulated Lung Fluid (SLF) Preparation

Simulated lung fluid was prepared as described by Marques *et al.* [1]. In particular, as SLF was selected Gamble's solution that represents the interstitial fluid within the lung. When preparing Gamble's solution, the components should be added in the order presented in Table S1 to avoid salt precipitation. Citrate was used instead of proteins to avoid foaming and acetate instead of organic acids. Gamble's solution has a pH of 7.4.

Table S2. Simulated Lung Fluid composition.

Components	Concentration (g/L)
Magnesium chloride ($MgCl_2$)	0.095
Sodium chloride (NaCl)	6.019
Potassium chloride (KCl)	0.298
Disodium hydrogen phosphate (Na_2HPO_4)	0.126
Sodium sulfate (Na_2SO_4)	0.063
Calcium chloride dihydrate ($CaCl_2 \cdot 2H_2O$)	0.368
Sodium acetate (CH_3COONa)	0.574
Sodium hydrogen carbonate ($NaHCO_3$)	2.604
Sodium citrate dihydrate ($Na_3C_6H_5O_7 \cdot 2H_2O$)	0.097

HPLC method details

Table S3. Summary of HPLC method details adopted for the API quantification.

API	HPLC Column	UV Wavelength (nm)	Mobile Phase	Flow rate (ml/min)	Injection volume (µl)	Column temperature (°C)
NCE-A	Atlantis dC18, 3.9x150 mm, 3.0 µm (Waters Corporation)	228	Gradient elution (Table S4). 0.02 M of NaH_2PO_4, (pH of 3.0 with H_3PO_4) and acetonitrile.	1	50	40
NCE-B	Atlantis dC18, 3.9x150 mm, 3.0 µm (Waters Corporation)	225	Gradient elution (Table S5). 0.02 M of NaH_2PO_4, (pH of 3.0 with H_3PO_4) and acetonitrile	1	50	40
Tobramycin	µBondapak® C18 3.9x300 mm, 10 µm (Waters Corporation)	360	Isocratic elution Tris (Hidroxymethyl) aminomethane, Sulfuric acid 1N and acetonitrile	1.2	20	25
Tiotropium Bromide	XB-C8 4.6x100 mm, 5 µm (Phenomenex®)	237	Isocratic elution Sodium Octane Sulfonate (pH of 3.2 with H_3PO_4) and acetonitrile	1	100	30
Indacaterol Maleate	Purospher® STAR RP-18 endcapped 5µm, 4 x 125 mm (Merk)	250	Isocratic elution KH_2PO_4 (pH of 4 with H_3PO_4) and acetonitrile	0.8	100	35

Table S4. Elution gradient of NCE-A.

Time (minutes)	NaH_2PO_4(%)	Acetonitrile (%)
0	40	60
2	20	80
5	40	60
6	40	60

Table S5. Elution gradient of NCE-B.

Time (minutes)	NaH_2PO_4(%)	Acetonitrile (%)
0	65	35
7	65	35
7.5	30	70
9.5	30	70
10	65	35
12	65	35

References

1. Marques MRC, Loebenberg R, Almukainzi M. Simulated Biological Fluids with Possible Application in Dissolution Testing. Dissolut Technol. 2011;18:15–28.
